# A predictive hemodynamic model based on risk factors for ruptured mirror aneurysms

**DOI:** 10.3389/fneur.2022.998557

**Published:** 2022-09-09

**Authors:** Sheng-qi Hu, Ru-dong Chen, Wei-dong Xu, Hua Li, Jia-sheng Yu

**Affiliations:** Department of Neurosurgery, Tongji Hospital, Tongji Medical College, Huazhong University of Science and Technology, Wuhan, China

**Keywords:** mirror aneurysm, risk factors, wall shear stress gradient ratio, combined hemodynamic parameter, predictive model, low shear area

## Abstract

**Objectives:**

To identify hemodynamic risk factors for intracranial aneurysm rupture and establish a predictive model to aid evaluation.

**Methods:**

We analyzed the hemodynamic parameters of 91 pairs of ruptured mirror aneurysms. A conditional univariate analysis was used for the continuous variables. A conditional multivariate logistic regression analysis was performed to identify the independent risk factors. Differences where *p* < 0.05 were statistically significant. A predictive model was established based on independent risk factors. Odds ratios (ORs) were used to score points. The validation cohort consisted of 189 aneurysms. Receiver operating characteristic curves were generated to determine the cutoff values and area under the curves (AUCs) of the predictive model and independent risk factors.

**Results:**

The conditional multivariate logistic analysis showed that the low shear area (LSA) (OR = 70.322, *p* = 0.044, CI = 1.112–4,445.256), mean combined hemodynamic parameter (CHP) (>0.087) (OR = 3.171, *p* = 0.034, CI = 1.089–9.236), and wall shear stress gradient (WSSG) ratio (>893.180) (OR = 5.740, *p* = 0.003, CI = 1.950–16.898) were independent risk factors. A prediction model was established: 23^*^LSA + 1^*^CHP mean (>0.087: yes = 1, no = 0) + 2 ^*^ WSSG ratio (>893.180: yes = 1, no = 0). The AUC values of the predictive model, LSA, mean CHP (>0.087), and WSSG ratio (>893.180) were 0.748, 0.700, 0.654, and 0.703, respectively. The predictive model and LSA cutoff values were 1.283 and 0.016, respectively. In the validation cohort, the predictive model, LSA, CHP (>0.087), and WSSG ratio (>893.180) were 0.736, 0.702, 0.689, and 0.706, respectively.

**Conclusions:**

LSA, CHP (>0.087), and WSSG ratio (>893.180) were independent risk factors for aneurysm rupture. Our predictive model could aid practical evaluation.

## Introduction

Intracranial aneurysms (IAs) are potentially fatal, and rupture is associated with dismal outcomes (1). Appropriate treatment is controversial because of treatment-related complications (e.g., thromboembolic events and intraoperative rupture) (2, 3). The indications for treatment can be controlled only when the risk of aneurysm rupture is adequately identified. Therefore, it is critical to identify aneurysms prone to rupture.

Computer fluid dynamics (CFD) simulations reflect the hemodynamics in IAs and numerically quantify rupture risks (4). It required accurate technical procedures, such as reliable 3D rotational angiography and boundary conditions (5, 6). Han et al. reported the significance of the average wall shear stress (WSS), oscillatory shear index (OSI), and Low Shear area in the 900 aneurysms (4). Zhang et al. derived novel flowrate-independent parameters that had better performance to predict rupture than above conventional parameters (7). Several rupture risk studies still yielded inconsistent results (8, 9). Although studies carried out technically accurate procedures (6), different patient-related factors, including gender, smoking, hypertension, hyperlipemia, and others, may cause selection bias and otherwise alter the results (3, 4, 10).

To prevent the influence of patient-related factors, we analyzed hemodynamic variables of mirror aneurysms using paired analysis. Previous studies only tested the accuracy of hemodynamic risk factors within their limited model cohorts (8, 9, 11). Thus, we aimed to comprehensively explore the hemodynamic risk factors and establish a valid predictive model.

## Methods

### Patient and data

**Figure 1:**
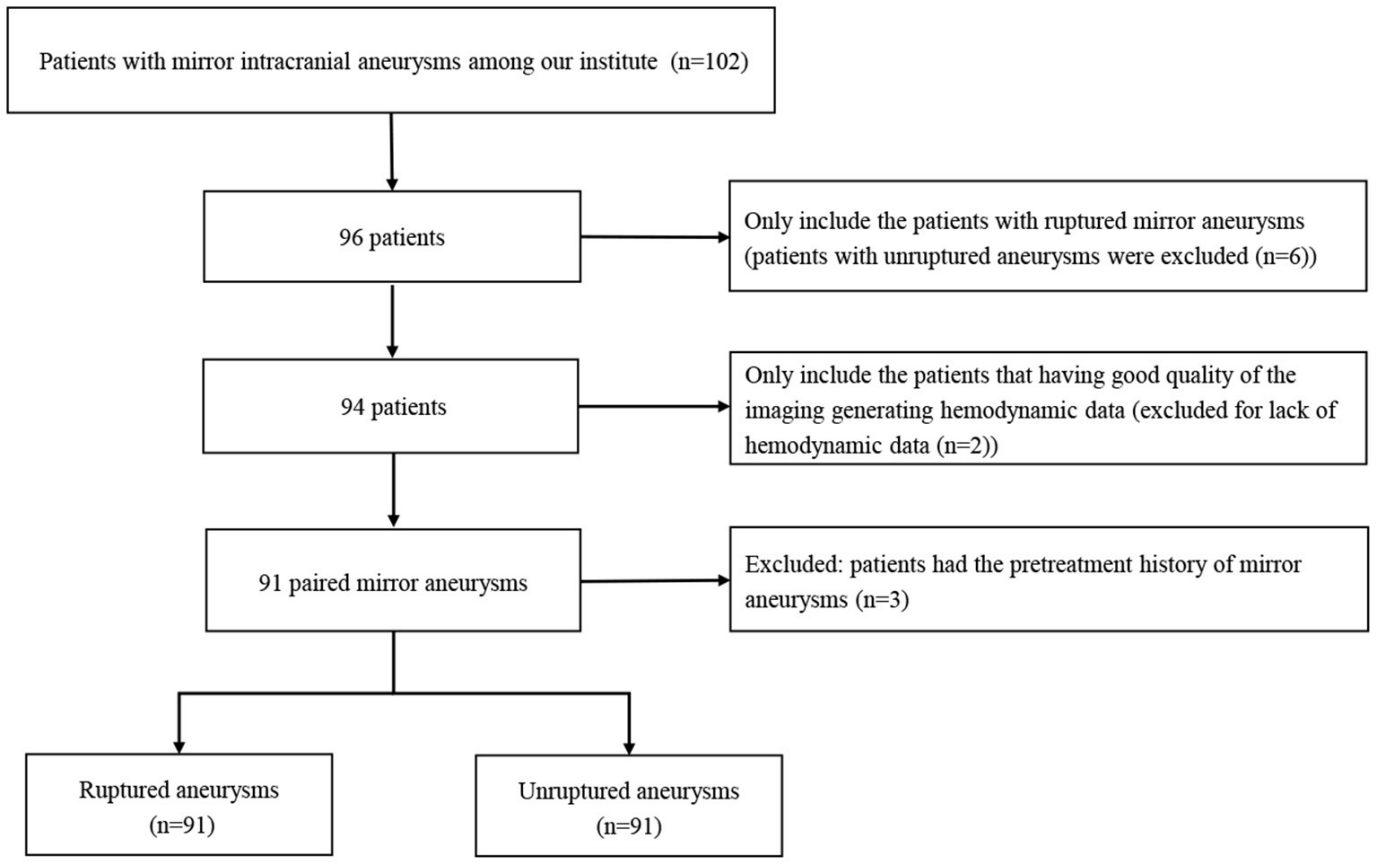
The flow chart of patient selection.

The flow chart of patient selection is shown in Figure 1. Our hospital's institutional ethics committee approved this study, and we collected data after obtaining the consent of the patients or their close relatives. From March 2012 to May 2022, 102 patients with mirror aneurysms underwent digital subtraction angiography (DSA) at our institute. Exclusion criteria were as follows: (1) unruptured mirror aneurysms (*n* = 6); (2) poor quality of the angiography leading to a lack of hemodynamic data (*n* = 2); (3) Pretreatment history of any aneurysm (*n* = 3). These 91 pairs of mirror aneurysms were divided into the ruptured group (*n* = 91) and the unruptured group (*n* = 91). In the modeling cohort, patients with SAH underwent emergent computed tomography and DSA within 72 h after hemorrhage. All ruptured aneurysms were treated by endovascular therapy or surgery promptly. Seventy-six unruptured aneurysms subsequently underwent therapy, and 10 insisted on annual follow-up. Five patients were lost to follow-up.

We collected the data of 189 patients diagnosed with single aneurysms admitted to our institute between May 2020 and May 2022. We acquired the consent of each patient in the validation cohort to undergo hemodynamic analysis. Their results were induced to verify the value of the prediction model.

### Hemodynamic variable calculation

**Figure 2:**
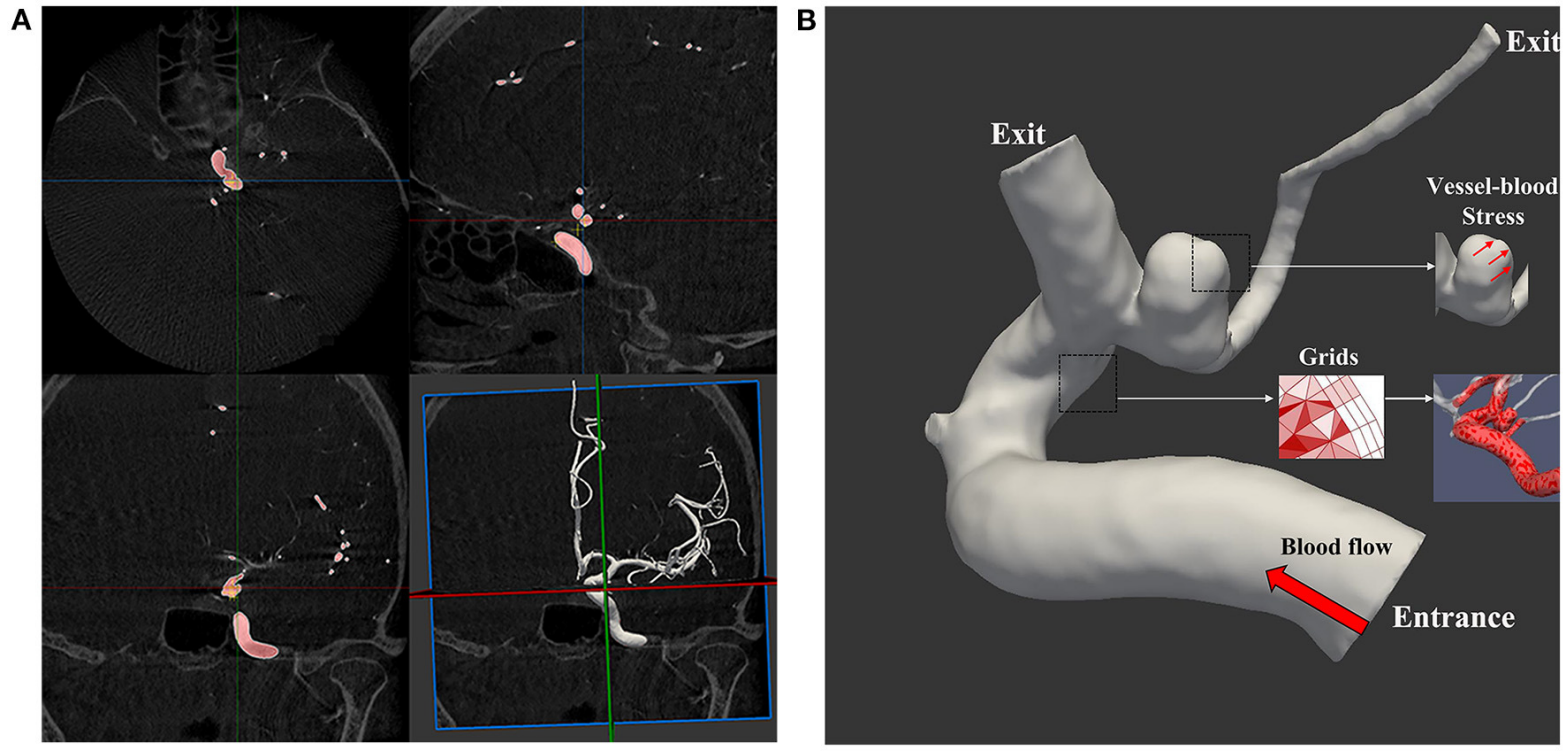
The process of the computational fluid dynamics simulation. **(A)** Three-dimensional digital subtraction angiography DICOM data import and vessel reconstruction; **(B)** set the relevant parameters, entrance and exit, and grids.

CFD simulations are presented in Figures 2A,B. The open-source CFD software package OpenFoam was used for grid generation and CFD calculation. Then three-dimensional DSA data were imported into the system. Mesh Generation: We OpenFoam to generation volume mesh. We used a mesh size of 0.1 mm for the inlet, outlet, and sac parts and a mesh size of 0.3 mm for the parent artery and other parts. Three layers of prism elements for the wall were used for the CFD simulations. The number of finite-volume grid elements used in this study was ~1 million. After mesh generation, the vascular model and blood flow state were generated using the following assumptions: the vascular wall was rigid, and the blood flow was isothermal and laminar (the blood was assumed to be an incompressible Newtonian fluid) (12). The blood flow was approximated using the unsteady Navier-Stokes equations. Blood density was set to 1,060 kg/m^3^, and blood viscosity was set to 0.004 N s/m^2^. Because the individual-specific blood flow conditions cannot be obtained in real-time, the standard data of the normal population obtained from the literature were used as the input boundary conditions (13), and the outlet was set at zero pressure (14). Finally, the calculation duration was set to last for three cardiac cycles. The results of the third cycle reached a stable state. Each cycle was set with 100 time steps. The results from the third simulated cardiac cycle were collected as output for the final analyses.

The hemodynamic variables in our study were described as follows: WSS was defined as the frictional force on the arterial wall produced by the blood flow in a direction toward a local tangent plane. $WSS=\frac{1}{T}\int_{0}^{T}|WSS_{i}|dt$; *WSS*_*i*_: an instantaneous WSS vector; T: the cycle duration (15, 16). Normalized wall shear stress (NWSS) was defined as the ratio of aneurysm WSS to the parent vessel's average WSS (17). Wall shear stress gradient (WSSG) was defined as the amplitude of variation along the wall shear force direction; $WSSG=\sqrt{{\left(\frac{\partial \tau_{w,p}}{\partial p}\right)}^{2}+{\left(\frac{\partial \tau_{w,q}}{\partial q}\right)}^{2}}$; τ_ω_, the WSS vector; the p-direction corresponds to the time-averaged direction the WSS, and the q-direction is perpendicular to the *p* (18, 19). Oscillatory shear index (OSI) was defined as the directional changes of WSS during the cardiac cycle; $OSI={\frac{1}{2}}^{*}\left(1-\frac{\left|\int_{0}^{T}wss_{i}dt\right|}{\int_{0}^{T}|wss_{i}|dt}\right)$ (16). Relative residence time (RRT) was defined as the residence time of particles near the wall; $RRT=\frac{1}{{\left(1-2^{*}OSI\right)}^{*}\frac{1}{T}\int_{0}^{T}|WSS_{i}|dt;}=\frac{1}{\frac{1}{T}|\int_{0}^{T}WSSidt|}$ (5). The combined hemodynamic parameter (CHP) was defined as the weighted average of WSS and OSI. $CHP=\omega_{1}^{*}\left(WSS_{norm}\right)+\omega_{2}^{*}\left(OSI_{norm}\right);\;\omega_{1}+\omega_{2}=1;\;{\left(WSS_{norm}-1\right)}^{4}+{\left(\frac{WSS-WSS_{\max}}{WSS_{\max}-WSS_{\min}}\right)}^{4}=1;\;OSI_{norm}=2^{*}OSI$ (16). The low shear area (LSA) was defined as the areas of the aneurysm wall exposed to a WSS below 10% of the mean parent vessel WSS, then normalized by the dome area (20). Ratio was defined as the maximum values divided by minimum values.

We calculated the minimum, maximum, mean, and ratio of WSS, NWSS, WSSG, OSI, RRT, and CHP. The LSA was calculated separately. All results of continuous variables were accurate to three decimal places.

### Statistical analysis

Statistical analysis was performed using SPSS 20.0 (IBM Inc., Chicago, IL). Continuous variables were expressed as median ± interquartile range. Categorical variables were expressed as frequencies (percentages). The Kolmogorov-Smirnov test was performed to determine whether the parameter dataset was normally distributed. A conditional univariate logistic analysis was used for the continuous variables. A conditional multivariate logistic regression was performed using the stepwise forward method to identify the independent risk factors. A predictive model was established based on the independent risk factors. Odds ratios (ORs) were used to allocate the predictive scores. Receiver operating characteristic (ROC) curves corresponding to the independent risk factors and the predictive model were generated to derive their respective areas under the curves (AUCs) and cutoff values. ROC curves were generated to validate the performance of the predictive model. The Delong test was performed to compare the AUCs. Differences where *p* < 0.05 were statistically significant.

## Results

### Patient demographics

We included 91 patients with mirror aneurysms in the model cohort and 189 patients with single aneurysms in the validation cohorts. In the model cohorts, 91 patients included 52 females and 39 males with a mean age of 58.10 years (41–83 years). In the validation cohorts, 189 patients included 104 females and 85 males with a mean age of 52.40 years (34–78 years). Demographics are displayed in Table 1.

**Table 1:** Demographic data in the model cohorts and validation cohorts.

**Demographic data**	**Model cohorts value**	**Validation cohorts value**
Total	91	189
Age	58.10 (41–83)	52.40 (34–78)
**Gender**		
Male	39 (42.86%)	85 (44.97%)
Female	52 (57.14%)	104 (55.03%)
**Location**		
MCA	44 (48.35%)	87 (46.03%)
PcoA	40 (43.96%)	64 (33.86%)
ACA	1 (1.10%)	3 (1.59%)
ICA bifurcation	1 (1.10%)	2 (1.06%)
Ophthalmic segment	2 (2.20%)	10 (5.29%)
AchA	1 (1.10%)	3 (1.59%)
VA	1 (1.10%)	5 (2.65%)
PICA	1 (1.10%)	1 (0.53%)
BA	0	6 (3.17%)
AcoA	0	8 (4.23%)
**Rupture site**		
Right	48 (52.75%)	107 (56.61%)
Left	43 (47.25%)	82 (43.39%)
**Hunt-Hess grades**		
I	10 (10.99%)	30 (15.87%)
II	55 (60.44%)	64 (33.86%)
III	14 (15.38%)	81 (42.86%)
IV	12 (13.19%)	12 (6.35%)
V	0	2 (1.06%)
Hypertension	58 (63.74%)	98 (51.85%)
Hyperlipidemia	32 (35.16%)	52 (27.51%)
Smoking	29 (31.87%)	47 (24.87%)
Drinking	20 (21.98%)	32 (16.93%)
Heart disease	15 (16.48%)	22 (11.64%)

MCA, middle cerebral artery; PcoA, posterior communicating artery; ACA, anterior cerebral artery; ICA, internal carotid artery; AcoA, anterior communicating artery; BA, basilar artery; AchA, anterior choroidal artery; VA, vertebral artery; PICA, posterior inferior cerebellar artery.

### Hemodynamic analysis

**Figure 3:**
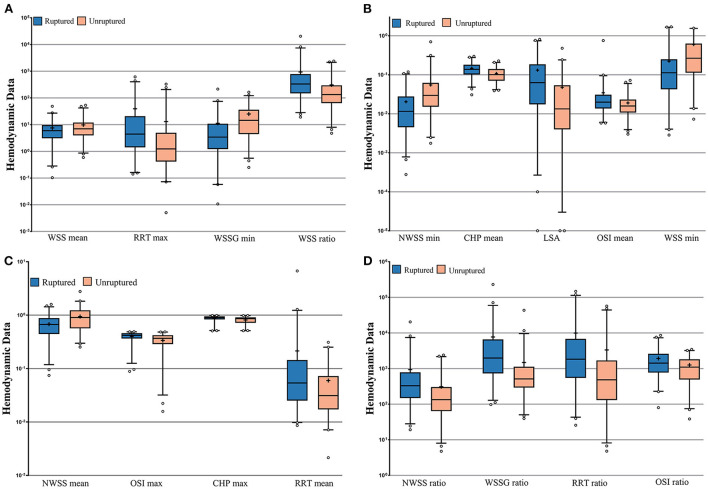
**(A–D)** Nested box plots showing the significant variables between the ruptured and unruptured roups. +: mean value; WSS, wall shear stress; NWSS, normalized wall shear stress; WSSG, wall shear stress gradient; OSI, oscillatory shear index; CHP, combined hemodynamic parameters; RRT, relative residence time; LSA, low shear area.

The results of the calculation and univariate analysis are presented in Table 2. The distribution of the significant variables is shown in Figure 3. The univariate logistic regression showed that compared to the unruptured group, ruptured aneurysms had significantly greater WSS ratio (*p* = 0.010), NWSS ratio (*p* = 0.011), WSSG ratio (*p* = 0.004), maximum OSI (*p* = 0.001), mean OSI (*p* = 0.004), OSI ratio (*p* = 0.003), maximum CHP (*p* = 0.005), mean CHP (*p* < 0.001), maximum RRT (*p* = 0.047), mean RRT (*p* = 0.005), RRT ratio (*p* = 0.042), and LSA (*p* = 0.001). The minimum WSS (*p* = 0.003), mean WSS (*p* = 0.048), minimum NWSS (*p* = 0.001), mean NWSS (*p* < 0.001), and minimum WSSG (*p* = 0.005) were significantly smaller in the ruptured group. The thresholds to discriminate the higher risk of an aneurysm with cutoff values of the hemodynamic factors with the highest sensitivity and specificity are displayed in Table 2.

**Table 2:** Measurement and analysis of the hemodynamics.

**Variables**	**Ruptured (*n* = 91)**	**Unruptured (*n* = 91)**	***p-value***	**Cutoff value**
**WSS**				
Min	0.113 ± 0.208	0.266 ± 0.520	0.003	0.259
Max	34.143 ± 28.774	32.827 ± 171.407	0.454	
Mean	5.950 ± 6.544	6.964 ± 8.073	0.048	6.417
Ratio	330.839 ± 632.763	133.596 ± 232.752	0.010	247.783
**NWSS**				
Min	0.012 ± 0.023	0.030 ± 0.047	0.001	0.013
Max	3.787 ± 1.871	4.043 ± 1.837	0.972	
Mean	0.669 ± 0.435	0.900 ± 0.665	<0.001	0.926
Ratio	330.840 ± 632.751	133.596 ± 238.152	0.011	177.219
**WSSG**				
Min	3.404 ± 9.597	14.478 ± 31.665	0.005	3.708
Max	6,554.117 ± 8,247.413	5,976.740 ± 9,065.646	0.826	
Mean	673.116 ± 850.285	828.386 ± 1,086.759	0.065	
Ratio	1,971.549 ± 5,849.403	512.353 ± 832.010	0.004	893.180
**OSI**				
Min	0.0002 ± 0.0003	0.0003 ± 0.0003	0.909	
Max	0.419 ± 0.093	0.369 ± 0.135	0.001	0.378
Mean	0.020 ± 0.017	0.016 ± 0.012	0.004	0.013
Ratio	1,426.591 ± 1834.415	1,098.070 ± 1,330.190	0.003	1,956.640
**CHP**				
Min	0.001 ± 0.001	0.001 ± 0.001	0.559	
Max	0.900 ± 0.119	0.855 ± 0.192	0.005	0.895
Mean	0.138 ± 0.079	0.101 ± 0.071	<0.001	0.087
Ratio	637.680 ± 602.570	577.055 ± 677.818	0.636	
**RRT**				
Min	0.003 ± 0.003	0.003 ± 0.003	0.677	
Max	4.428 ± 19.238	1.233 ± 4.542	0.047	1.120
Mean	0.054 ± 0.119	0.031 ± 0.055	0.005	0.023
Ratio	1,833.052 ± 6,263.718	483.643 ± 1,559.518	0.042	535.906
**LSA**	0.063 ± 0.168	0.013 ± 0.050	0.001	0.016

WSS, wall shear stress; NWSS, normalized wall shear stress; WSSG, wall shear stress gradient; OSI, oscillatory shear index; CHP, combined hemodynamic parameter; RRT, relative residence time; LSA, low shear area.

**Figure 4:**
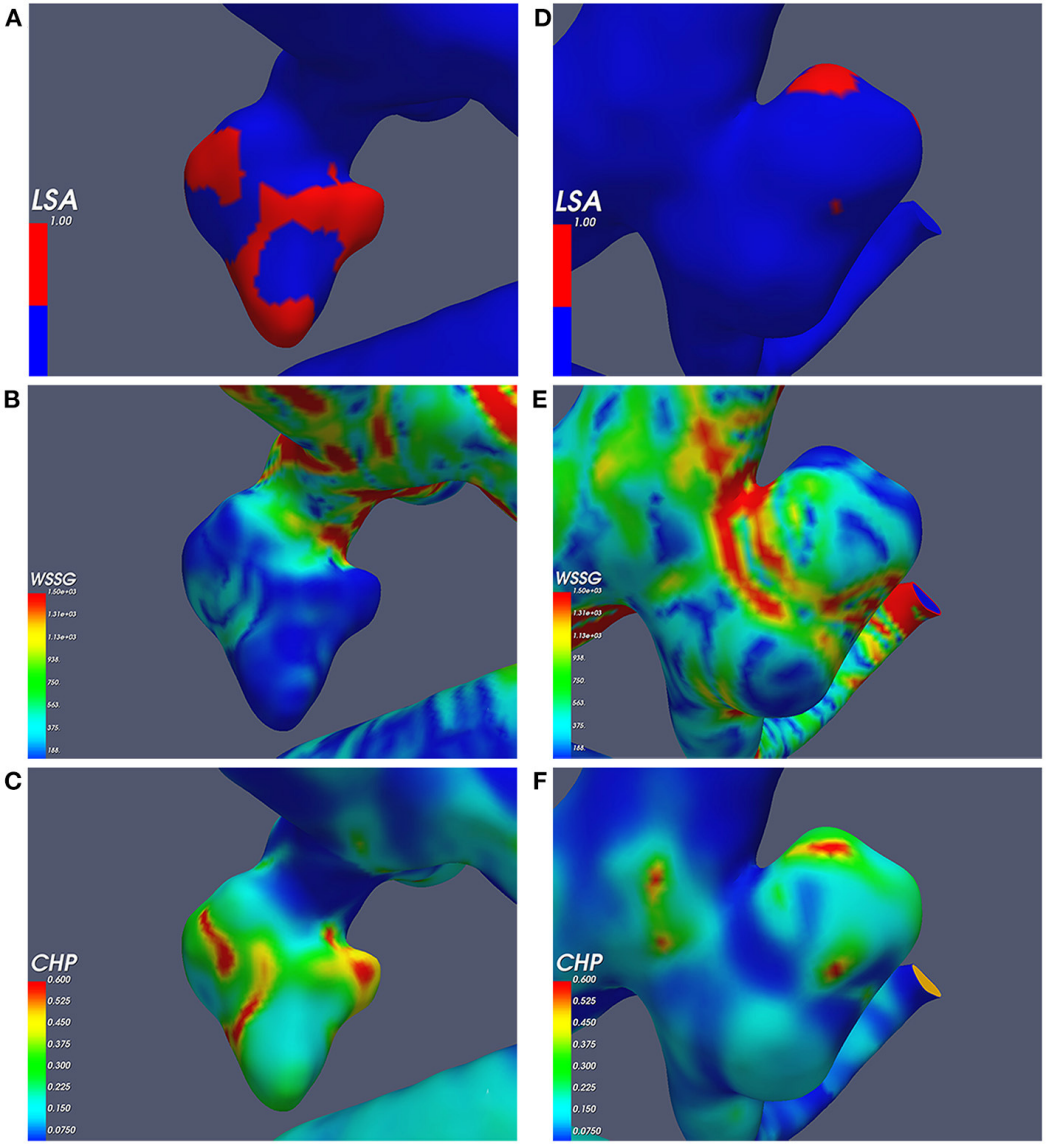
Independent risk factors; **(A–C)** the ruptured aneurysm's wall shear stress gradient (WSSG), combined hemodynamic parameter (CHP), and low shear area (LSA); **(D–F)** the unruptured aneurysm's WSSG, CHP, and LSA.

The conditional multivariate logistic regression analysis is shown in Table 3. The LSA (OR = 70.322, *p* = 0.044, CI = 1.112–4,445.256), mean CHP (>0.087) (OR = 3.171, *p* = 0.034, CI = 1.089–0.236), and WSSG ratio (>893.180) (OR = 5.740, *p* = 0.003, CI = 1.950–16.898) were independent risk factors. The differences in the independent risk factors are shown in Figure 4.

**Table 3:** The result of conditional multivariable logistic regression.

**Variables**	**β-value**	***p-value***	**Odds ratio**	**Confidence interval**	**Predictive score**
LSA	4.253	0.044	70.322	1.112–4,445.256	23
Mean CHP (>0.087)	1.154	0.034	3.171	1.089–9.236	1
WSSG ratio (>893.180)	1.747	0.002	5.740	1.950–16.898	2

LSA, low shear area; CHP, combined hemodynamic parameter; WSSG ratio: wall shear stress gradient ratio.

**Figure 5:**
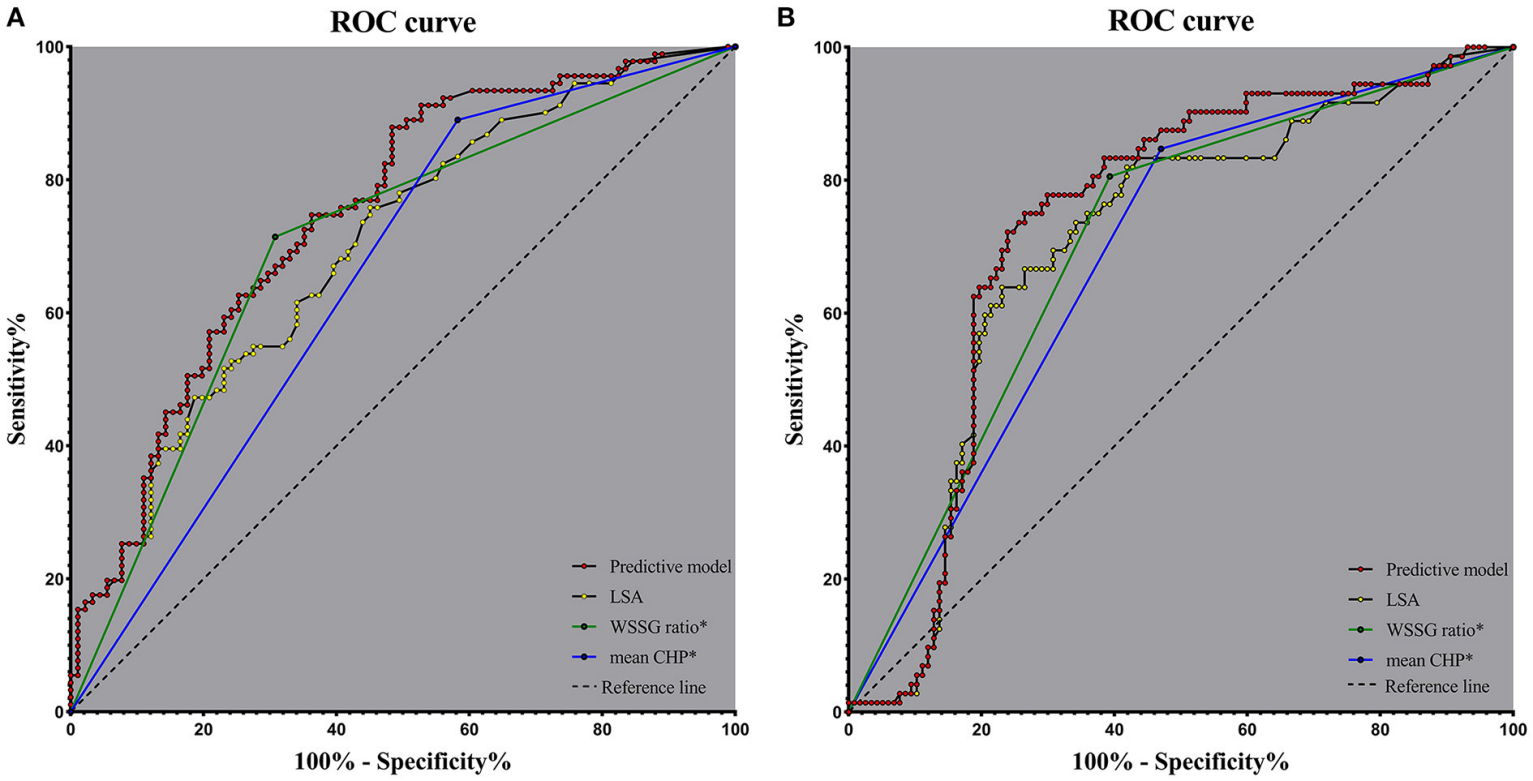
Receiver operating characteristics curve analysis in the model cohort **(A)** and validation cohort **(B)**; The predictive model based on the LSA, mean CHP (>0.087), and WSSG ratio (>893.180). mean CHP*: mean CHP (>0.087), WSSG ratio*: WSSG ratio (>893.180); CHP, combined hemodynamic parameter; WSSG, wall shear stress gradient; LSA, low shear area.

We generated a predictive model based on the independent risk factors. OR values were allocated to the predictive scores. We assigned the LSA, mean CHP (>0.087), and WSSG ratio (>893.180) as 23, 1, and two points, respectively, as shown in Table 3. The predictive model was as follows: 23^*^LSA + 1^*^ mean CHP (>0.087: yes = 1, no = 0) + 2 ^*^ WSSG ratio (>893.180: yes = 1, no = 0). The AUC values of the predictive model, LSA, mean CHP (>0.087), and WSSG ratio (>893.180) were 0.748, 0.700, 0.654, and 0.703, respectively. The ROC analysis is presented in Figure 5A. The predictive model and LSA cutoff values were 1.283 and 0.016, respectively. The AUC of the predictive model was significantly greater than the LSA (Delong test, *p* = 0.006, Cl = 0.014–0.081), mean CHP (>0.087) (Delong test, *p* = 0.001, CI = 0.037–0.151), and WSSG ratio (>893.180) (Delong test, *p* = 0.034, CI = 0.003–0.085).

We also calculated the ROCs in the validation cohorts of 189 IAs to verify the value of the predictive model and independent risk factors. The AUC values of the predictive model, LSA, mean CHP (>0.087), and WSSG ratio (>893.180) were 0.736, 0.702, 0.689, and 0.706, respectively (Figure 5B).

## Discussion

Hemodynamic studies of aneurysm rupture require attention. CFD simulation is based on reliable 3D rotational angiography, which reconstructs the shape of the parent vessel and IA (6). Previous studies included the morphologic and hemodynamic variables, and we found the former outweighed the latter; hemodynamics was even insignificant (11, 21). Schisterman et al. found that strategies ignoring causal structures lead to biased effect estimations (22). Qiu et al. demonstrated that morphology affects the distribution and magnitude of WSS (20). The correlation may decrease the significance of hemodynamics. Furthermore, hemodynamic studies in IA rupture yielded inconsistent results (8–10, 18). One reason may be attributable to differences in patient-related factors. The study of mirror aneurysms can avoid the influence of modifiable inter-individual variations and reflect actual risk factors associated with rupture (3). However, previous studies of mirror aneurysms are limited because of small sample sizes, and they do not have the appropriate hemodynamic models to evaluate the risk (8, 9, 11, 23). In the present study, the LSA, mean CHP (>0.087), and WSSG ratio (>893.180) were independent risk factors. Furthermore, a predictive model was established based on three independent risk factors that showed good predictive performance (AUC = 0.748). Our multivariate results and predictive model demonstrated the role of hemodynamics in prediction.

### Mean CHP

CHP combines the WSS and the OSI. Previous studies focused on the role of WSS and OSI on the growth or the rupture and returned inconsistent results (4, 9, 10, 15). Xiang et al. found that low WSS and high OSI were related to the rupture in a study of 119 IAs (24). However, that study was limited by the small sample size and individual differences (24). CHP combined the WSS with the OSI and supported their findings. The study of Miura et al. stressed the significance of lower WSS and higher OSI, and the mean values were close to ours (WSS, 7.19 vs. 7.52; OSI, 0.016 vs. 0.018) in univariate analysis; however, the OSI was not independent (25). Our study classified the mean CHP exceeding the optimal cutoff value of 0.870 as a higher risk. Mean CHP (>0.087) was as an independent risk factor, highlighted by its risk of rupture (OR = 3.171, *p* = 0.034, CI = 1.089–9.236). This finding demonstrates that the risk of rupture increases 3.171-fold, as the mean CHP exceeded 0.087.

Cho et al. stressed that the CHP reflected the thin-walled area, which was the primary location of the rupture (16). The thin-walled area parallels the high CHP. Jiang et al. demonstrated that the thinner wall has a lower WSS and higher pressure (17). Mean CHP represents the average level in the aneurysm, and a higher mean CHP suggests a higher risk. Recent studies did not offer the normal reference values for CHP. Our findings suggest that a mean CHP of more than 0.087 helps identify the threshold for aneurysm rupture.

### WSSG ratio

WSSG is a spatial concept that indicates whether the increasing WSS occurred in an accelerating or decelerating flow. The consensus states that WSSG positively correlates with rupture (19, 26). Positive WSSG is associated with endothelial migration, apoptosis, and aneurysmal extracellular remodeling (27). Degradation of the elastic constituents is driven by deviations of the WSSG from normotensive values (28). Wei et al. reported an average WSSG value close to ours (730.568 vs. 673.116); nevertheless, that study lacked extreme data to analyze the extent of deviations (18). Zhai et al. reported that the mean value of ruptured aneurysms' maximum and minimum WSSG were 7,295.350 and 5.029, respectively. The ratio (1,483.787) exceeded our threshold (893.180) and was accompanied by a high risk of rupture (29). The WSSG ratio combines extreme values to demonstrate the amplitude of variation. The WSSG ratio, as a ratio between the maximum and minimum, has not been noted in previous studies. Specific values are needed to discriminate risks. Our study found that the risk of rupture would increase 5.740-fold as the WSSG ratio exceeded 893.180. WSSG ratio (>893.180) is a two-category variable that applies a risk threshold and practical predictive means.

### LSA

The LSA reflects the area of the low WSS in aneurysms. In our study, the LSA was an independent risk factor (OR = 70.322, *p* = 0.044, CI = 1.112–4,445.256) with a cutoff value of 0.016. We included the LSA in the predictive model to determine the positive correlation between the LSA with the rupture. Our results were in favor of the low WSS theory. Low and stagnant flows cause flow-induced inflammation and degradation of the aneurysm wall (30). Qiu et al. of the study treating 72 IAs demonstrated that ruptured aneurysms' median values were higher than unruptured IAs (0.09 vs. 0.02) (20). A nationwide matched case-control study demonstrated this significant risk whose mean value is close to ours (0.153 vs. 0.131) (31); however, it did not provide an OR value or cutoff values to discriminate the risk of rupture. In the present study, 0.016 was the rupture threshold, which aids valuable prediction. In addition, because the LSA ranges from 0 to 1, a large OR does not mask the role of other independent risk factors. These findings suggest that combinations of parameters are needed, as demonstrated by our prediction model.

### Predictive model

We established a predictive model based on the independent risk factors. Our combination model out-performed any single variable (0.748 vs. 0.700, 0.654, and 0.703, respectively). Although the OR values generated accurate predictions, clinical application is hampered by inconvenience. We used approximated and simplified values. The LSA, mean CHP (>0.087), and WSSG ratio (>893.180) were scored as 23, 1, and two points, respectively. These three factors can be summarized directly to identify aneurysms with the highest risk of rupture.

A predictive study by Detmer et al. identified population-specific differences that determine the association between hemodynamics and rupture risk (32). Our model using mirror aneurysms avoided the influence of patient-related factors. CFD was based on the simulation of the flow in aneurysms, parent vessels, and morphology that significantly impact the hemodynamics (11). Several studies involving morphology underestimated some hemodynamic parameters (20, 33). By contrast, our predictive is clinically applicable. The cutoff value of our predictive score was 1.283, which could aid practical evaluations.

### Limitations

This study has some limitations. First, retrospective studies cannot determine causality between features and outcomes. Second, because of the rarity of mirror aneurysms at a single institution, the limited sample size might have reduced the robustness of our findings. Third, the Newtonian fluid assumption could influence the low WSS role on rupture (34). This study used generalized boundary conditions and properties of blood similar to the study of Ford et al. (13). It might not reflect the patient-specific conditions. Some studies revealed that different inlet and outlet boundary conditions lead to differences in the results (5) so that the inflow and outflow conditions were adjusted for each aneurysm (7). Therefore, it is crucial to define the boundary conditions carefully for each case. Future work will include the collection of patient-specific flow rates and the usage of the splitting method to determine the outflow rates. Finally, although our results are generalizable to aneurysms in most locations, mirror middle cerebral artery and posterior communicating artery aneurysms account for a large proportion. In future studies, more cases should be gathered at several centers to identify other independent factors and increase the predictive model's accuracy.

## Conclusions

In this retrospective study, we analyzed mirror aneurysms to avoid the influence of inter-individual differences and aneurysm locations. We found that hemodynamic analysis helps predict aneurysm rupture risks. The LSA, mean CHP (>0.087), and WSSG ratio (>893.180) were independent risk factors for aneurysm rupture. Our predictive model based on three independent risk factors aids practical evaluation.

## Data availability statement

The raw data supporting the conclusions of this article will be made available by the authors, without undue reservation.

## Ethics statement

The study was reviewed and approved by the institutional ethics committee of Tongji Hospital. Written informed consent was obtained from the patients or their close relatives.

## Author contributions

S-qH and R-dC: conceptualized the study, wrote the manuscript, and collected and analyzed data. HL and W-dX: revised the draft paper. J-sY: supervision. All authors contributed to the article and approved the submitted version.

## Conflict of interest

The authors declare that the research was conducted in the absence of any commercial or financial relationships that could be construed as a potential conflict of interest.

## Publisher's note

All claims expressed in this article are solely those of the authors and do not necessarily represent those of their affiliated organizations, or those of the publisher, the editors and the reviewers. Any product that may be evaluated in this article, or claim that may be made by its manufacturer, is not guaranteed or endorsed by the publisher.
